# Country, Sex, EDSS Change and Therapy Choice Independently Predict Treatment Discontinuation in Multiple Sclerosis and Clinically Isolated Syndrome

**DOI:** 10.1371/journal.pone.0038661

**Published:** 2012-06-29

**Authors:** Claire Meyniel, Timothy Spelman, Vilija G. Jokubaitis, Maria Trojano, Guillermo Izquierdo, François Grand’Maison, Celia Oreja-Guevara, Cavit Boz, Alessandra Lugaresi, Marc Girard, Pierre Grammond, Gerardo Iuliano, Marcela Fiol, Jose Antonio Cabrera-Gomez, Ricardo Fernandez-Bolanos, Giorgio Giuliani, Jeannette Lechner-Scott, Edgardo Cristiano, Joseph Herbert, Tatjana Petkovska-Boskova, Roberto Bergamaschi, Vincent van Pesch, Fraser Moore, Norbert Vella, Mark Slee, Vetere Santiago, Michael Barnett, Eva Havrdova, Carolyn Young, Carmen-Adella Sirbu, Mary Tanner, Michelle Rutherford, Helmut Butzkueven, on behalf of the MSBasis Study Group

**Affiliations:** 1 Department of Neurology, Royal Melbourne Hospital, Victoria, Australia; 2 CHU Nantes, CIC 0004, Nantes, France; 3 University of Bari, Bari, Italy; 4 Hospital Universitario, Sevilla, Spain; 5 Clinique Neuro Rive-Sud, Greenfield Park, Quebec, Canada; 6 University Hospital La Paz, IdiPAZ, Madrid, Spain; 7 Karadeniz Technical University, Trabzon, Turkey; 8 MS Center, Department of Neuroscience and Imaging, University “G. d’Annunzio”, Chieti, Italy; 9 Hotel-Dieu de Levis, Department of Neurology, Levis, Quebec, Canada; 10 Ospedali Riuniti di Salerno, Salerno, Italy; 11 FLENI, Buenos Aires, Argentina; 12 Centro Internacional de Restauracion Neurologica, Havana, Cuba; 13 Hospital Universitario Virgen de Valme, Seville, Spain; 14 Ospedale di Macerata, Macerata, Italy; 15 John Hunter Hospital, Newcastle, New South Wales, Australia; 16 Hospital Italiano, Buenos Aires, Argentina; 17 New York University Hospital for Joint Diseases, New York, New York, United States of America; 18 Clinic of Neurology Clinical Center, Skopje, Macedonia; 19 Neurological Institute IRCCS Mondino, Pavia, Italy; 20 Cliniques Universitaires Saint-Luc, Brussels, Belgium; 21 Jewish General Hospital, Montreal, Canada; 22 Mater Dei Hospital, Msida, Malta; 23 Flinders University and Medical Centre, Adelaide, South Australia, Australia; 24 HIGA Gral, San Martin La Plata, Argentina; 25 Brain Mind Research Institute, Camperdown, New South Wales, Australia; 26 General Teaching Hospital, Prague, Czech Republic; 27 The Walton Centre for Neurology and Neurosurgery, Liverpool, United Kingdom; 28 Central University Emergency Military Hospital, Bucharest, Romania; 29 Department of Medicine, Melbourne Brain Centre, The University of Melbourne, Victoria, Australia; 30 Department of Neurology, Box Hill Hospital, Monash University, Melbourne, Victoria, Australia; Julius-Maximilians-Universität Würzburg, Germany

## Abstract

**Objectives:**

We conducted a prospective study, MSBASIS, to assess factors leading to first treatment discontinuation in patients with a clinically isolated syndrome (CIS) and early relapsing-remitting multiple sclerosis (RRMS).

**Methods:**

The MSBASIS Study, conducted by MSBase Study Group members, enrols patients seen from CIS onset, reporting baseline demographics, cerebral magnetic resonance imaging (MRI) features and Expanded Disability Status Scale (EDSS) scores. Follow-up visits report relapses, EDSS scores, and the start and end dates of MS-specific therapies. We performed a multivariable survival analysis to determine factors within this dataset that predict first treatment discontinuation.

**Results:**

A total of 2314 CIS patients from 44 centres were followed for a median of 2.7 years, during which time 1247 commenced immunomodulatory drug (IMD) treatment. Ninety percent initiated IMD after a diagnosis of MS was confirmed, and 10% while still in CIS status. Over 40% of these patients stopped their first IMD during the observation period. Females were more likely to cease medication than males (HR 1.36, p = 0.003). Patients treated in Australia were twice as likely to cease their first IMD than patients treated in Spain (HR 1.98, p = 0.001). Increasing EDSS was associated with higher rate of IMD cessation (HR 1.21 per EDSS unit, p<0.001), and intramuscular interferon-β-1a (HR 1.38, p = 0.028) and subcutaneous interferon-β-1a (HR 1.45, p = 0.012) had higher rates of discontinuation than glatiramer acetate, although this varied widely in different countries. Onset cerebral MRI features, age, time to treatment initiation or relapse on treatment were not associated with IMD cessation.

**Conclusion:**

In this multivariable survival analysis, female sex, country of residence, EDSS change and IMD choice independently predicted time to first IMD cessation.

## Introduction

The first attack of multiple sclerosis (MS), commonly an optic neuritis, a transverse myelitis or a brainstem syndrome, is known as a clinically isolated syndrome (CIS). Randomised-placebo controlled studies of immunomodulatory drugs (IMDs) in patients with CIS and early relapsing-remitting MS (RRMS) report a significant decrease in relapse rate and a reduction of brain lesion accumulation, suggesting that IMD therapy should be introduced at an early stage of the disease [1]–[7].

As IMD therapies are only partially effective and are parenterally administered, treatment persistence is a major issue. It has been reported by one large study that only 55% of MS patients continue their IMD for an 18-month period [8]. Other studies have reported IMD discontinuation rates between 2% and 20% during the first 6 months and up to 67% at 1 year [9]–[13]. Even though IMD discontinuation is a frequent occurrence, factors leading to treatment cessation are not well known. To our knowledge, no previous studies were designed to prospectively follow patients in clinical practice from disease onset to assess factors that could predict IMD discontinuation.

This prospective cohort study aims to characterise treatment persistence and the predictors of treatment discontinuation in CIS and in early RRMS, specifically focussing on the first IMD treatment initiated.

## Methods

### Ethics Statement

Human research ethics committee approval or waivers and written informed consent from patients were obtained at each participating site.

### Database

The MSBase Incident Study (MSBASIS) is a world-wide, investigator-initiated observational cohort study of patients with CIS. The study commenced in December 2004. Data for the current analysis was extracted on the 7^th^ of February 2011. This cohort study enrols patients from 44 MS treatment centres in 17 countries: Italy, Spain, Canada, Australia, The Netherlands, Argentina, France, Turkey, Denmark, Cuba, Macedonia, The USA, Belgium, Malta, Czech Republic, The UK and Romania. For the present analysis, only centres with more than 10 enrolled cases were included. The MSBASIS study is a sub-study of the international MSBase Registry [14], a strictly observational prospective cohort study monitoring routine clinical care of people with MS attending outpatient clinics in MS specialist centres. Minimum datasets of MS-related outcomes are updated at least annually within the registry. Data of patients meeting the inclusion criteria is collected by physicians using iMed, an electronic patient record system, which generates anonymised data extract files that are uploaded to the MSBase registry. Quality assurance through online certification of Expanded Disability Status Scale (EDSS) competency is required at each participating site.

### Study Procedures

Patients were eligible for the study if their CIS was diagnosed or confirmed by a participating neurologist and a baseline visit was completed within 12 months of CIS onset. Patients with primary progressive MS were excluded from this analysis. Minimum baseline data requirements included: the date of CIS onset and clinical presentation, a neurological evaluation including Kurtzke Functional System (KFS) scores together with an EDSS score, and a cerebral magnetic resonance imaging (MRI) scan available at baseline (within 12 months of CIS onset). The first cerebral MRI after onset of CIS was evaluated according to the Barkhof criteria for dissemination in space [15]. Other diagnostic test information was recorded if performed, including spinal MRI lesion number and the presence of CSF-restricted oligoclonal banding. Following this initial visit, minimum annual follow-up was required, although all follow-up visits were recorded. The minimum data collected at follow up were the date of visit, KFS, EDSS, the date of onset and duration of relapses, glucocorticoid therapy to treat relapses, and commencement and cessation of disease-modifying drugs. Participating investigators used iMed as their clinic management tool and entered required study data at the time of clinic visits. Incomplete or missing datasets were monitored and followed-up bi-annually by the central study coordinator.

### Definitions

The KFS and EDSS scores were determined according to the Neurostatus system [16]. Baseline cerebral MRI scans were performed with or without gadolinium contrast administration. If performed with gadolinium administration, the presence or absence of contrast-enhancing lesions was recorded. For this analysis, the number of cerebral T2 lesions was categorised in 2 groups as 0–8 lesions or 9+ lesions. EDSS change during treatment was defined as the EDSS difference between the earliest and latest recorded visits within the treated observation period.

### Statistical Analysis

Sex, relapse, MRI criteria, clinic location and IMD product were summarised using frequencies and percentages. Age, change in EDSS and symptom duration were assessed for skew and as all demonstrated non-normality these were described using medians and inter-quartile ranges (IQR). Survival analysis provided discontinuation estimates at different time points. Kaplan-Meier estimates were used to describe the cumulative probability of first treatment discontinuation. Cox Proportional Hazards Regression was used to model associations between MRI criteria, change in EDSS, IMD product and relapses with time to treatment discontinuation, adjusted for potential confounders identified a priori, including age at treatment commencement, symptom duration, sex and clinic location (Table 1). Hazard proportionality was assessed by analysis of scaled Schoenfeld residuals. In the multivariable analysis illustrated in Table 2, due to highly discordant IMD product discontinuation rates in different countries, analysis of Italy as a separate location category led to violation of hazard proportionality. We were able to resolve this only through combining the Italian cohort with the cohort from “other” countries, which then permitted derivation of a valid proportional adjusted Cox model. We derived and tested for interaction effects between location and other predictors within the multivariable model including DMT with no statistically significant interactions demonstrated. All reported p values are two-tailed and for each analysis p<0.05 was considered significant. All analyses were performed using Stata version 11.0 (StataCorp, College Station, Texas).

**Table 1:** Characteristics of the CIS and RRMS patients treated by IMD.

		All IMD	IM IFNβ-1a	SC IFNβ-1a	IFNβ-1b	GA
**Patients (n)**		1247	362	440	251	194
**Age at treatment commencement (years) - median (IQR)**		32.2 (26.0, 39.9)	33.2 (26.5, 39.9)	30.8 (24.8, 38.6)	32.3 (27.0, 41.7)	33.8 (28.2, 40.6)
**Years between onset of symptoms and treatment start** **(years) - median (IQR)**		0.7 (0.4, 1.1)	0.7 (0.4, 1.1)	0.7 (0.4, 1.1)	0.7 (0.4, 1.3)	0.7 (0.4, 1.2)
**Change in EDSS - median (IQR)**		0 (−0.5, 0.5)	0 (−0.5, 0.5)	0 (−1.0, 0.5)	0 (−0.5, 0.5)	0 (−1.0, 0)
**Sex - n (%)**	Female	892 (71.5)	257 (71.0)	305 (69.3)	181 (72.1)	149 (76.8)
**Location - n (%)**	Australia	165 (13.2)	35 (9.7)	41 (9.3)	57 (22.7)	32 (16.5)
	Canada	208 (16.7)	67 (18.5)	71 (16.1)	37 (14.7)	33 (17.0)
	Netherlands	164 13.2)	28 (7.7)	66 (15.0)	42 (16.7)	28 (14.4)
	Spain	119 (9.5)	35 (9.7)	31 (7.1)	30 (12.0)	23 (11.9)
	Italy	273 (21.9)	92 (25.4)	130 (30.0)	16 (6.4)	35 (18.0)
	Other	318 (25.5)	105 (29.0)	101 (23.0)	69 (27.5)	43 (22.2)
**At least one relapse during treatment n (%)**		507 (40.7)	142 (39.2)	202 (45.9)	89 (35.5)	74 (38.1)
**MRI - n (%)**	9+ T2 hyperintensive lesions	422 (33.8)	118 (32.6)	134 (30.5)	98 (39.0)	72 (37.1)
	At least 1 gadolinium enhancing lesion*	256 (25.5)	55 (19.9)	114 (30.7)	50 (25.3)	37 (23.3)
	Gadolinium injection not done	243 (19.5)	86 (23.8)	69 (15.7)	53 (21.1)	35 (18.0)

Abbreviations: IM: intramuscular, SC: subcutaneous, IFN: interferon, GA: glatiramer acetate.

**Table 2:** Predictors of discontinuation of immunomodulatory treatments in CIS an early MS (Cox Regression).

Predictors	Level	Discontinuations (% of level)	Incidence rate per 100 person-years (95% CI)	Unadjusted		Adjusted**		
				HR (95% CI)	p	HR (95% CI)	p	
**Age at treatment commencement**	−	−	−	0.99 (0.98, 1.00)	***0.047***			
**Years between onset of symptoms and treatment start**	−	−	−	0.97 (0.88, 1.07)	0.543			
**Change in EDSS**	−	−	−	1.21 (1.14, 1.28)	***<0.001***	1.21 (1.14, 1.28)	***<0.001***	
**Sex**	Female	42.7	18.52 (16.75, 20.48)	1.38 (1.13, 1.70)	***0.002***	1.36 (1.11, 1.68)	***0.003***	
	Male	34.4	13.04 (10.91, 15.58)	1.00		1.00		
**Location**	Australia	47.3	28.08 (22.49, 35.05)	1.80 (1.21, 2.68)	***0.004***	1.98 (1.33, 2.96)	***0.001***	
	Canada	51.4	21.01 (17.39, 25.40)	1.56 (1.06, 2.28)	***0.023***	1.50 (1.02, 2.21)	***0.038***	
	Netherlands	42.7	17.86 (14.13, 22.57)	1.29 (0.86, 1.94)	0.218	1.30 (0.87, 1.96)	0.206	
	Spain	29.4	14.93 (10.72, 20.79)	1.00		1.00		
	Italy*	38.1	14.04 (11.59, 17.02)	1.03 (0.70, 1.51)	0.873			
	Other	34.3	13.49 (11.79, 15.43)	0.97 (0.67, 1.43)	0.993	0.99 (0.69, 1.42)	0.971	
**At least one relapse during treatment**	Yes	50.7	19.70 (17.44, 22.27)	1.37 (1.15, 1.63)	***<0.001***			
	No	33.2	14.57 (12.86, 16.52)	1.00				
**MRI - T2 hyperintensive lesions**	<9	39.8	16.49 (14.70, 18.50)	1.00				
	9+	41.0	18.04 (15.54, 20.93)	1.09 (0.90, 1.32)	0.366			
**MRI - Gadolinium enhancing lesion**	Zero	40.2	16.64 (14.86, 18.63)	1.00				
	At least 1	41.0	17.86 (14.75, 21.63)	1.06 (0.85, 1.32)	0.613			
	Not done	39.9	16.32 (13.37, 19.91)	1.01 (0.81, 1.32)	0.911			
**Treatment**	IM IFNβ-1a	43.9	17.89 (15.31, 20.90)	1.38 (1.02, 1.85)	***0.035***	1.40 (1.04, 1.89)	***0.028***	
	SC IFNβ-1a	43.2	18.80 (16.31, 21.67)	1.45 (1.09, 1.94)	***0.012***	1.50 (1.12, 2.01)	***0.006***	
	IFNβ-1b	37.5	15.13 (12.35, 18.54)	1.16 (0.84, 1.60)	0.381	1.07 (0.77, 1.48)	0.679	
	GA	30.9	12.74 (9.89, 16.40)	1.00		1.00		

Abbreviations: CI: Confidence Interval, HR: Hazard Ratio, IM: intramuscular, SC: subcutaneous, IFN: interferon, GA: glatiramer acetate.

* Italy combined with ‘Other’ in multivariable (adjusted) analysis to satisfy hazard proportionality assumption.

** Analysis of scaled Schoenfeld residuals: test of proportional hazards, p = 0.07.

## Results

### Study Sample

A total of 2314 patients (70.9% female and 29.1% male) with CIS were included in MSBASIS by their physicians and followed for a median duration of 2.7 years (IQR 1.42, 4.69). Median age at the onset of the disease was 31.5 (IQR 25.1, 39.0) years. The main locations in which patients were recruited were Italy (22.7%), Canada (15.0%), Australia (10.9%), Spain (10.5%) and The Netherlands (10.4%). Baseline brain MRI scans were performed a median of 68 days from CIS onset. During the follow-up period, 1271 patients (55.9%) experienced at least one relapse.

### Treatment Characteristics

A total of 1247 patients received an IMD, of which 125 patients (10%) were initially treated as CIS patients and 1094 (88%) had converted to RRMS at the time of their first IMD commencement. Two percent of patients had a secondary progressive MS at the time of their first treatment initiation. There was a median time of 8.4 months (IQR 4.8, 13.2 months) between the onset of first symptoms and initial IMD start. Median EDSS at treatment onset was 2 (IQR 1.0, 2.5). A total of 362 patients received intramuscular (IM) interferon-beta (IFNβ)-1a, 440 received subcutaneous (SC) IFNβ-1a, 251 received IFNβ-1b and 194 received glatiramer acetate (GA). Two hundred and seventy three patients treated with an IMD lived in Italy, 208 in Canada, 165 in Australia, 164 in The Netherlands, 119 in Spain and 318 in other countries. Cerebral MRI was performed with Gadolinium injection in 80.5% of the patients. Baseline characteristics for patients treated with each IMD treatment are reported in Table 1. Investigator-reported reason for treatment discontinuation was not a mandatory part of the dataset, but was reported in three broad categories for 35% of the discontinuations. Adverse event/patient choice was reported for 18.7% of cessations, disease progression and lack of benefit for 10.5% and scheduled stops (eg pregnancy) for 5.2%.

### Predictors of Discontinuation of First IMD

Table 2 summarises the univariable and multivariable analyses of predictors of discontinuation in the entire cohort. The overall rate of discontinuation was 40.3% for all the IMD treatments combined. Unadjusted Cox-regression modelling identified baseline characteristics of female sex, age at start of treatment, country of residence (increased rates of discontinuations in Australia and Canada) and type of treatment (increased rates of discontinuation with the two IFNβ-1a preparations) as significantly associated with increased rates of discontinuation. Additionally, both EDSS increase and relapse during treatment were also associated with increased cessation rates.

**Figure 1:**
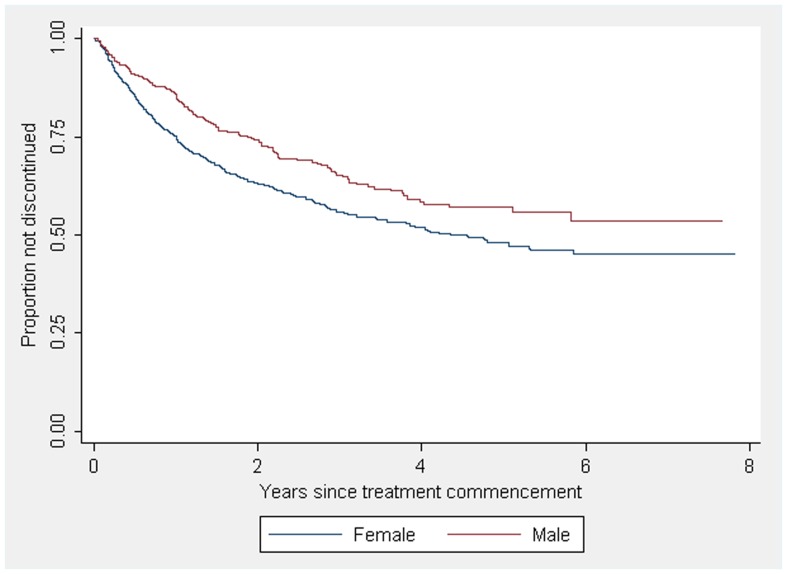
Kaplan-Meier survival estimates for treatment discontinuation by patient sex in CIS an early MS. Figure 1 demonstrates that female sex is associated with a higher IMD discontinuation rate compared to male sex in our prospectively followed multinational, multicentre cohort.

In the multivariable analysis, sex, location, change in EDSS and IMD product identity were all independently predictive of treatment discontinuation (Table 2). Females ceased immunomodulatory treatment at a higher rate compared to males (Figure 1). Patients in Australia and in Canada experienced increased rates of first IMD discontinuation compared to patients managed in Spain. The third significant predictor of IMD cessation was an increase in EDSS score during treatment. The Hazard ratio of treatment cessation was 1.21 per unit increase in EDSS score during treatment. Although predictive of increased cessation rates on univariable analysis, occurrence of a relapse on treatment and age at treatment commencement were not significant in multivariable analyses, regardless of the combination of covariates it was modelled with, and were thus excluded from the final multivariable model.

**Figure 2:**
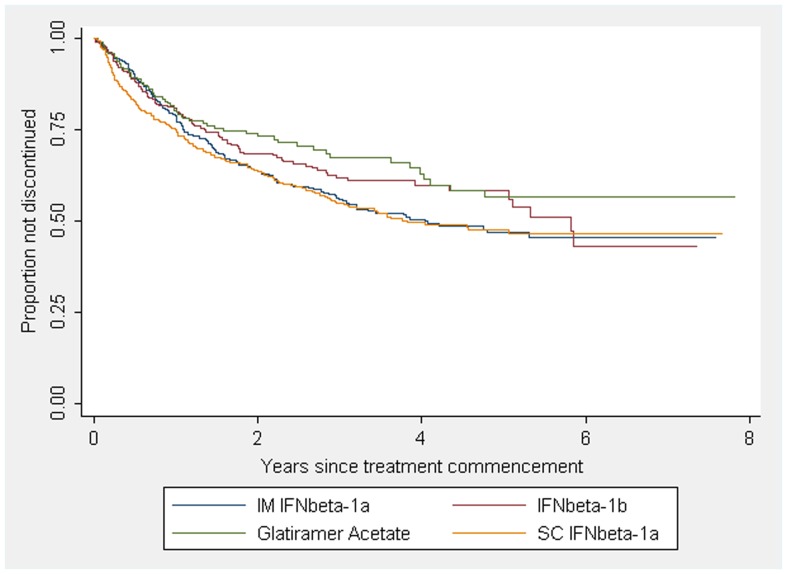
Kaplan-Meier survival estimates for first treatment discontinuation by IMD in CIS an early MS. Figure 2 demonstrates a greater rate of IMD discontinuation in early RRMS and CIS populations prescribed IM IFNbeta-1a and SC IFNbeta-1a as compared with IFNbeta-1b and Glatiramer Acetate.

The IMD product identity was also associated with different discontinuation rates in the adjusted analysis (Figure 2). After commencement of IM IFNβ-1a, 44% of the patients, followed up for a median of 3.0 years, ceased their treatment. For SC IFNβ-1a, there were 43% of discontinuations over a 2.9 year median follow-up time. For IFNβ-1b, there were 37% discontinuations over 2.8 years of median follow-up time and 31% of the patient treated with GA stopped their treatment over a 2.3 year median follow-up period. In the multivariable analysis, patients whose first IMD was one of the two IFNβ-1a preparations had significantly higher discontinuation rates than those who commenced on IFNβ-1b or GA. However, IMD discontinuation rates for each product varied greatly between different countries (see Table 3).

**Table 3:** Predictors of discontinuation by immunomodulatory treatment in early MS and CIS (Univariable Cox Regression).

Predictor	Level	IM IFNβ-1a				SC IFNβ-1a		IFNβ-1b			GA		
		UHR (95% CI)	p			UHR (95% CI)	p	UHR (95% CI)		p	UHR (95% CI)		p
**Age at treatment commencement**	−	0.98 (0.97, 1.00)	0.059			0.99 (0.97, 1.00)	0.086	1.00 (0.98, 1.02)		0.760	1.02 (1.00, 1.05)		0.105
**Years between onset of symptoms** **and treatment start**	-	0.99 (0.84, 1.18)	0.935			0.98 (0.81, 1.18)	0.848	0.93 (0.78, 1.11)		0.410	1.07 (0.84, 1.37)		0.584
**Change in EDSS**	-	1.23 (1.12, 1.36)	***<0.001***			1.26 (1.14, 1.40)	***<0.001***	1.01 (0.87, 1.19)		0.857	1.24 (1.03, 1.50)		***0.021***
**Sex**	Female	1.45 (1.00, 2.08)	***0.048***			1.49 (1.07, 2.07)	***0.018***	1.52 (0.93, 2.47)		0.094	0.94 (0.52, 1.69)		0.842
	Male	1.00				1.00		1.00			1.00		
**Location**	Australia	2.58 (1.23, 5.43)			***0.013***	1.42 (0.62, 3.27)	0.409	1.52 (0.77, 3.02)		0.229	2.57 (0.84, 7.92)		0.099
	Canada	2.40 (1.23, 4.68)			***0.010***	1.51 (0.70, 3.24)	0.289	0.95 (0.45, 2.02)		0.901	1.54 (0.50, 4.79)		0.455
	Netherlands	1.05 (0.46, 2.44)			0.901	1.25 (0.57, 2.76)	0.577	0.96 (0.46, 2.00)		0.908	2.92 (0.96, 8.89)		0.059
	Spain	1.00				1.00		1.00			1.00		
	Italy	1.40 (0.72, 2.73)			0.322	1.01 (0.48, 2.13)	0.974	0.46 (0.15, 1.43)		0.180	0.83 (0.25, 2.76)		0.759
	Other	1.05 (0.53, 2.06)			0.890	1.14 (0.55, 2.35)	0.719	0.53 (0.24, 1.12)		0.068	0.94 (0.29, 3.07)		0.925
**At least one relapse during treatment**	Yes	1.61 (1.18, 2.20)			***0.003***	1.15 (0.86, 1.52)	0.352	1.49 (0.99, 2.24)		0.057	1.24 (0.75, 2.06)		0.407
	No	1.00				1.00		1.00			1.00		
**MRI - T2 hyperintensive lesions**	<9	1.00				1.00		1.00			1.00		
	9+	1.06 (0.76, 1.49)			0.718	1.18 (0.87, 1.61)	0.287	0.85 (0.55, 1.31)		0.461	1.52 (0.89, 2.59)		0.126
**MRI - Gadolinium enhancing lesion**	At least 1	1.16 (0.75, 1.79)			0.505	1.12 (0.81, 1.55)	0.494	1.03 (0.61, 1.75)		0.898	0.59 (0.27, 1.33)		0.203
	Zero	1.00				1.00		1.00			1.00		
	Not done	1.13 (0.78, 1.64)			0.516	0.87 (0.57, 1.33)	0.518	0.99 (0.59, 1.66)		0.962	1.17 (0.62, 2.18)		0.631

Abbreviations: CI: Confidence Interval, UHR: Unadjusted Hazard Ratio, IM: intramuscular, SC: subcutaneous, IFN: interferon.

GA: glatiramer acetate.

Baseline cerebral MRI characteristics (presence of gadolinium enhanced lesion or number of T2 lesions) and the time between CIS onset and treatment commencement were not associated with time to treatment discontinuation.

### Predictors of Discontinuation for Each Immunomodulatory Treatment

In the univariable analysis of the predictors of discontinuation for each of the four IMDs, female sex was found to be a significant predictor of increased rate of discontinuation of IM and SC IFNβ-1a, with a strong trend for IFNβ-1b (Table 3). However, female sex was not a risk factor for discontinuation in GA-treated patients. Whereas increasing disability was a significant predictor of discontinuation for IM and SC IFNβ-1a and GA, relapse occurrence on treatment was a significant factor of discontinuation only for patients treated with IM IFNβ-1a. Discontinuation rates for IMDs were greater in Canada (51.4%) and Australia (47.3%) than in Italy (38.1%) or Spain (29.4%), with marked differences in discontinuation hazards between IMD preparations within countries (Table 3). For instance, relative to Spain as a comparator, the Australian patients’ discontinuation rates were much higher for IM IFNβ-1a and for GA than for SC IFNβ-1a and IFNβ-1b.

Age at treatment commencement and MRI features, namely gadolinium enhancing lesion presence and T2 hyperintense lesion load, were not associated with discontinuation rate for any of the IMDs.

### Treatment Decisions Post Discontinuation of Immunomodulatory Treatment

Of the 503 recorded first IMD discontinuations, 9 patients did not record a subsequent EDSS visit and a further 53 patients recorded visits less than 12 months post-discontinuation follow-up (as of the data extract date). Of the remaining 441 patients who discontinued treatment, 301 (68.3%) commenced a second IMD within 12 months of ceasing the first. Of these 301 patients, only 6.9% restarted on their original IMD. Median time to start a second IMD in this group was 5 days (IQR: 0, 45). Conversely, almost one third of patients (31.7%) did not recommence an IMD within twelve months of first IMD cessation.

## Discussion

The objective of this study was to identify factors influencing first IMD discontinuation in early RRMS and CIS during up to 6 years of follow up. Our overall rate of discontinuation was 40.3%, with a median study follow-up of 2.7 years. Of these patients, 31.7% remained untreated 12 months after IMD discontinuation. Female sex, country of residence, EDSS change on treatment and IMD choice were the most important independent factors influencing time to first IMD discontinuation. Baseline MRI criteria did not have a significant influence on treatment persistence.

Although data gathered in prospective cohort studies is less complete than that generated by clinical trials, there are also advantages of analyses such as the one presented [17]. Firstly, as our study is observational, it allows the characterisation of long-term drug utilisation patterns. The creation of a pre-defined study protocol, contemporaneous and prospective recruitment, centralised data monitoring and data query generation contribute to high data accuracy. Given that only patients seen within one year of their first reported symptom onset (median time of 4.5 months) are included, our dataset provides much greater data accuracy than retrospective studies, which are often confounded by recall bias. Focussing on discontinuations of first IMD only allows us to analyse discontinuation without the potential biases related to previous IMD exposure. Additionally, the nature of the MSBASIS data acquisition, in which the MSBase registry receives anonymised data uploads from an electronic medical record used in day-to-day clinical management, facilitates data accuracy and timeliness of event reporting because no separate transcription from medical records to online data portals or paper forms is required. Several countries host multicentre registry studies including Denmark, Sweden, Italy, Germany and France [18], [19]. However, MSBase is the first global online MS registry using a contemporaneously recruited study population assessed using the same protocol and thus allows us to compare persistence rates in different countries.

Previous studies have reported discontinuation rates in patients with RRMS [8]–[13]. In general, the discontinuation rates reported in other studies vary widely, possibly suggesting geographic trends. A retrospective study in British Colombia reported a 39% discontinuation rate during a 3-year mean follow-up period for patients treated with IFNβ and a high proportion of interruption (27%) the first 6 months [20]. In an Italian study, 46% of the patients stopped their treatment with IFNβ during a 4.2 year mean follow-up period [10]. However, two recent US studies found a much higher rate of discontinuation for patients treated with GA and IFNβ (45% over an 18 month period and between 40% and 67% at one year follow-up, respectively) [8], [12]. Both of these latter studies were retrospective and based on administrative claim data from insurance companies. Interestingly, a Spanish study reported a very low rate of IMD discontinuation (17% stopped and 5% switched their IMD during a 47 month mean time of follow up), very similar to data obtained in post-marketing studies [13], [21]. Our discontinuation rate of 40.3% with a median study follow-up of 2.7 years is in the mid-range of most of the other studies, with the exception of the Spanish study. To our knowledge, none of the previous studies have followed patients in clinical practice from disease onset.

The general definition of treatment adherence includes treatment compliance and treatment persistence. Compliance can be defined as the ability to follow a pre-specified administration schedule without missing doses, not assessed in the current study. Treatment persistence refers to a patient’s ongoing motivation to continue a given treatment. Discontinuation can be initiated by the physician or the patient, or, ideally by consensus between both. Reasons for patient-initiated and physician-initiated discontinuation could be different. Physicians could suggest discontinuation when they observe relapses or disability progression, whereas patients might cease an IMD unilaterally if they suffer severe injection related side effects, needle phobia or persistent flu-like symptoms, issues that they might be reluctant to discuss with their treating physician [22], [23]. The main reasons for IMD discontinuation identified in the literature to date are the ones suggested above, namely injection-related side effects, perceived lack of efficacy and adverse events [13], [20]. In an Italian study, only 4.9% of the patients stopped their IMD treatment for any other reason [10].

After an IMD treatment discontinuation, patients either switch to another IMD or disease-modifying drug or disengage from treatment. In either case, IMD discontinuation generally represents a failure of the initial prescribed therapy. Our study is the first to systematically assess the rate of first IMD discontinuation, among seen-from onset patients with CIS or early RRMS.

One key finding in this analysis is that, after adjusting for other factors, female sex is associated with increased rates of discontinuation in early MS and CIS, consistent with prior reports [8], [13], [20]. An increase of adverse effects such as flu-like symptoms with fixed dose interferon medications related to a low body mass index of female compared to male patients might be one explanation, [23] as we did not observe an excess of female discontinuations for GA. A second reason for female treatment discontinuation might be desire of a pregnancy.

Our study confirms that increasing EDSS score is a predictor of treatment discontinuation. This is consistent with prior registry studies, which identified relapses and EDSS instability as associated with treatment discontinuation [13], [20].

The global nature of our cohort reveals major regional differences. As mentioned above, different rates of discontinuation were reported in prior studies with higher rates in the United States and Canada compared with Spain [10], [13], [20]. As these studies were methodologically heterogeneous, they are difficult to compare. Our study clearly shows that persistence rates in study centres in Spain and Italy were much higher than in Australian or Canadian centres. This may relate to differences in patient attitudes and the therapeutic relationship in different countries. One hypothesis is that patients in Southern Europe are more strongly influenced by their doctors to persist with treatment than patients in Canada or Australia. Such cultural differences have previously been described between German and Italian populations [24], [25,]. Additionally, standardised prescription protocols for IMDs exist in Spain, which require frequent patient review [26]. This may lead to stronger doctor-patient interactions and lower discontinuation rates in Spain.

In contrast to an American and an Italian study reporting an increased discontinuation rate for patients treated with GA and IFNβ-1b compared to IM IFNβ-1a [12], [21], our cohort showed a decrease of discontinuation in patients treated with GA and IFNβ-1b compared to the IFNβ-1a products. Our study only includes one US centre and we did find large differences in product-specific discontinuation rates between different countries, so it is possible that these discordant results reflect different study populations.

The limitations of this study are the same as those that pertain to other prospective cohort studies, namely that data gathered in studies such as ours is less complete than that generated by clinical trials. In order to address this, a central study co-ordinator was employed to monitor data completeness and follow up with centres with incomplete records.

At the time of data extraction, detailed (categorical) reasons for treatment discontinuation were not obtained, however this will be redressed in the future.

The benefits of IMD treatment in early RRMS and CIS include reduction in relapse rates, disability progression and MRI activity [27]. Patients who cease IMD treatment are at greater risk of relapse activity and disability progression. A better understanding of the factors influencing higher discontinuation rates in women and the large geographical differences that we have characterised in this study could, in the future, allow us to improve management strategies for patients at high risk of discontinuation.
